# 小细胞肺癌驱动基因研究进展

**DOI:** 10.3779/j.issn.1009-3419.2016.04.10

**Published:** 2016-04-20

**Authors:** 静 赵

**Affiliations:** 100730 北京，中国医学科学院北京协和医院呼吸内科 Department of Respiratory Medicine, Peking Union Medical College Hospital, Chinese Academy of Medical Sciences, Beijing 100730, China

**Keywords:** 肺肿瘤, 小细胞肺癌, 驱动基因, Lung neoplasms, Small cell lung cancer, Driver mutations

## Abstract

小细胞肺癌是一类具有高度侵袭性的肺恶性肿瘤，预后极差，近30年来，其治疗策略无明显进展。积极研究小细胞肺癌分子生物学特征，并筛选潜在驱动基因，有助于为小细胞肺癌开拓新的治疗途径，改善疾病预后。本文将对小细胞肺癌驱动基因研究相关进展进行综述。

## Abstract (en)

Small cell lung cancer (SCLC) is an aggressive tumor and prognosis remains dismal. Screening for the targetable driver mutations of this malignant tumor contributes to improve the therapeutic approaches and outcome. This review will report the advances on some latest driver mutations.

肺癌发病率及死亡率均高居恶性肿瘤第一位，小细胞肺癌（small cell lung cancer, SCLC）约占肺癌总发病率10%-15%，其临床特点和生物学行为异于其他类型肺癌，表现为倍增时间短，早期易发生转移，恶性程度极高。目前SCLC患者的治疗仍以全身化疗±同步放疗为主，初治缓解率高，但复发率极高，且易发生耐药，5年生存率不足5%。

随着肿瘤分子生物学的迅速发展，针对表皮生长因子受体（epidermal growth factor receptor, *EGFR*）、间变性淋巴瘤激酶（anaplasticlymphoma kinase, *ALK*）突变基因的分子靶向药物在肺腺癌患者中取得了满意的疗效及生存获益，为非小细胞肺癌（non-small cell lung cancer, NSCLC）开辟了新的治疗途径。但SCLC的治疗始终无明显进展，迫切需要新的有效治疗手段。关于SCLC发生发展分子机制方面的各项研究进展相对缓慢，迄今尚未发现有效的分子治疗靶点。其中一个重要原因可能是，绝大多数SCLC患者在就诊时已发生远处转移，失去手术机会，无法获取足够的、令人满意的手术病理标本进行大规模突变基因筛选。

目前，综合已有的几项较大规模SCLC全基因组测序结果，已知潜在的SCLC分子生物治疗靶点主要包括抑癌基因*TP53*、*RB-1*、*PTEN*缺失或失活，PIK3CA、*EGFR*、*c-MET*、*c-Kit*过度表达，*FGFR1*、*SOX2*、*MYC*家族扩增，其他可检测到的还包括*EP300*、*CREBBP*、*ZNF521*、*RUNX1T1*、*KMT2D*等基因突变^[1-5]^。迄今为止，多种针对上述靶点的药物已经探索性地进入临床研究阶段，然而尚无一种靶向治疗能够得到有效的临床证实。2012年Byers等^[6]^报告了SCLC中两个较为独特的分子特征：果蝇*zeste*基因增强子的人类同源物-2（enhancer of zeste homolog 2, EZH2）和聚腺苷酸二磷酸核糖聚合酶-1[poly(ADP-ribose)polymerase, PARP-1]，提出二者作为SCLC潜在驱动基因，有可能为SCLC治疗开辟新的方向。

## 1 EZH2和PARP-1

Byers等^[6]^应用反向阶段蛋白质阵列技术，测定了34株SCLC细胞系及74株NSCLC细胞系共计193种蛋白表达水平情况。该研究发现，RTK、PI3K、RAS/MAP/ERK在SCLC中的活性以及表达水平远远低于NSCLC，但在SCLC中，转录基因*E2F1*调控因子如EZH2、DNA修复蛋白、胸苷酸合成酶、凋亡因子的表达水平明显高于NSCLC，尤其以EZH2和PARP-1的升高为主。敲除*EZH2*和*PARP-1*基因后，SCLC细胞系的生长受到明显抑制。

### 1.1 EZH2

RB1/E2F信号传导途径异常存在于超过90%的SCLC，是其公认的最具代表性的异常信号传导通路^[7]^。Coe等^[8]^对14份SCLC病理标本及14株SCLC细胞系进行基因检测，在全部标本中均检测到了RB1/E2F信号传导途径上的异常，包括*RB1*抑癌基因缺失或突变、E2F家族扩增。而无论是RB1丢失或E2F扩增，最终都将激活其下游的*EZH2*基因。与此同时，Coe等^[8]^研究了EZH2在SCLC细胞中的表达情况，结果显示*EZH2*基因的表达水平均有明显升高。*RB1*抑癌基因主要通过抑制E2F转录因子家族表达起到抑制细胞增殖作用，而E2F家族成员如E2F1在不同的人类癌症微环境中可能起到促进或抑制癌细胞增殖这两种截然相反的作用^[9]^。因此，存在于RB1/E2F信号传导途径下游*EZH2*基因似乎更适合作为SCLC驱动基因。

*EZH2*基因定位于人染色体7q35位点上，是多梳基因（polycomb group genes, *PcG*）家族重要成员之一。PcG家族是人体生长发育过程中一类重要表观遗传调控因子，家族成员间相互结合成多梳抑制复合体发挥作用，能使某些特定基因的沉默，在DNA修复中也起到一定作用^[10]^。EZH2是多梳抑制复合体2的关键催化亚单位，在染色质结构的形成、基因表达调节以及生长控制等过程中发挥重要作用。

*EZH2*基因异常激活后可以甲基化组蛋白3的27号赖氨酸（histone H3 lysine 27, H3K27），与DNA甲基转移酶（DNA methyltransferases, DNMTs）、组蛋白去乙酰化酶（histone deacetylases, HDACs）一起发挥转录抑制作用，沉默200多种抑癌基因的表达，亦可激活某些靶基因，导致恶性肿瘤形成发展^[11, 12]^。Hubaux等^[13]^利用短发夹RNA（short hairpin RNA, shRNA）沉默SCLC细胞系中的*EZH2*基因表达后，观察到处于S或G_2_/M期的细胞比例降低，p21蛋白水平升高，促凋亡因子Puma和Bad上调，细胞凋亡活性明显增加，认为这可能为EZH2在SCLC形成中的分子作用机制。

近年来，研究者们陆续在前列腺癌、乳腺癌、膀胱癌等多种肿瘤组织中发现*EZH2*基因高表达现象^[14-16]^。其中，对乳腺癌、前列腺癌的研究显示EZH2可能与肿瘤的高度侵袭性相关。有观点认为EZH2可以使某些抗转移基因表达沉默，如E-钙粘蛋白、基质金属蛋白酶组织抑制因子等。也有观点认为EZH2可参与潜在转移源头“肿瘤干细胞”的自我更新^[17]^。对卵巢癌的研究则表明EZH2与顺铂耐药性的产生密切相关^[18]^。目前，*EZH2*基因在癌变过程中的具体作用机制尚不十分清楚。目前关于EZH2抑制剂的研究仍在进行中，尚无成熟的EZH2抑制剂进入临床前期试验。

### 1.2 PARP-1

PARP超家族主要在碱基切除修复（base excision repair, BER）过程发挥重要作用，17个家族成员中以可以生成活性形式聚腺苷二磷酸核糖[poly（ADP-ribose）, PAR]的PARP-1、PARP-2、PARP5A、PARP5B研究较为深入透彻^[19]^，被认为是潜在的肿瘤生物治疗靶点。PARP-1是此家族中结构最典型、最为重要的一个异构体，是在DNA受损时最早出现并起主要修复作用的蛋白质之一^[20, 21]^。

PARP-1以尼克酰胺腺嘌呤二核苷酸为底物，生成PAR，后者可使RNA聚合酶、DNA聚合酶等多种DNA修复蛋白发生聚ADP核糖基化，显著增强其DNA修复能力，使整个DNA修复过程顺利完成。BER是DNA发生DNA单链断裂（single strand breaks, SSBs）时的主要修复途径，SSBs若得不到及时修复，可逐渐累积进展至更为严重的DNA双链断裂（double stand breaks, DSBs），后者修复主要依赖同源重组（homologous recombination, HR）和非同源性末端接合（non-homologous end joining, NHEJ）2种途径。抑癌基因*BRCA*是HR途径中的主要成员。PARP-1亦可通过HR及NHEJ途径参与DSBs修复。故在*BRCA*基因突变患者中，BER途径的DNA修复作用尤其必不可少。而反过来根据生物体内的“合成致死现象”（synthetic lethality），在BRAC功能缺失情况下抑制肿瘤细胞*PARP*基因的表达，DNA损伤将无法得到有效修复从而导致死亡。

Byers等^[6]^对135例肺癌标本进行了免疫组化分析，结果显示PARP-1在SCLC中的表达水平显著高于腺癌及鳞癌。体外试验中，NSCLC细胞系对PARP-1抑制剂均表现出了不同程度的抵抗性，然而，绝大多数SCLC细胞对PARP-1抑制剂如AZD2281、AG014699则较敏感。可能的机制是，SCLC作为一种乏氧肿瘤，其慢性缺氧环境可刺激E2F/p130结合抑癌基因*BRCA1*和*RAD5*启动子位点，导致BRCA1和RAD51表达水平下调。BRCA、PARP介导的DNA修复途径均被抑制，最终引起SCLC细胞凋亡^[22]^。在放疗或化疗诱导出现癌细胞SSBs损伤时，PARP抑制剂通过抑制DNA修复作用可进一步增强肿瘤细胞对放化疗敏感度。

目前，多种PARP抑制剂如rucaparib、niraparib、olaparib已进入Ⅲ期临床试验。针对SCLC的BMN673、viliparib已完成Ⅰ期临床试验，正在进行Ⅱ期临床试验。值得注意的是，Cardnell等^[23]^进行的一项研究发现伴有PI3K途径激活的SCLC细胞系往往对PARP抑制剂耐药。Juvekar等^[24]^在乳腺癌小鼠模型中观察到，联合应用PI3K抑制剂NVP-BKM120与PRAP抑制剂olaparib可延长肿瘤倍增时间达70 d以上，数倍于单用NVP-BKM120或olaparib的延长时间。上述研究结果提示，在SCLC治疗中，可尝试联用PI3K抑制剂与PRAP抑制剂，以改善PARP抑制剂敏感性，增强治疗效果。

## 2 其他传统驱动基因

### 2.1 P13K-AKT-mToR信号传递通路

P13K-AKT-mToR途径为EGFR、HER2等RTKs主要下游信号传递通路之一，该通路异常激活能够加速肿瘤细胞周期、抑制细胞凋亡、促进肿瘤细胞迁移，以及增加化疗药物耐药性。SCLC是最早报道存在该通路异常的众多肿瘤之一。

#### 2.1.1 PTEN

*PTEN*是抑癌基因，位于人染色体10q23位点上，负性调节PI3K下游AKt/mToR信号通路的活性。有研究^[25]^显示，大约15%的SCLC中存在*PTEN*基因突变，突变后的*PTEN*基因功能丧失，可引起第二信使PIP3的大量聚集，使得下游通路被激活，从而促进肿瘤的形成。

#### 2.1.2 PIK3CA

负责编码PI3Ks家族的催化亚单位p110a，在肿瘤细胞中，p110a主要作用为上调Akt的表达水平，并通过磷酸化多种底物，包括凋亡相关蛋白、mTOR组成成分等，促进细胞存活、生长和增殖。日本学者扩大了基因突变筛查范围后，在SCLC细胞株及SCLC病理标本中分别发现23%、13%的*PIK3CA*基因突变^[26]^。目前已知PI3K抑制剂如wortmannin和LY294002对携带*PIK3CA*突变的SCLC细胞有效^[27]^。问题是目前上述抑制剂均未表现出适当的靶向特异性，潜在毒副作用较大。

#### 2.1.3 mTOR

mTOR属于磷脂酰肌醇3-激酶相关激酶（plosphatidylinositol 3-kinase-related kinase, PIKK）蛋白家族的一员，活化的*mTOR*基因使蛋白翻译过程中的某些因子（最主要是4EBP1和P70S6K）发生磷酸化，进而参与多项细胞功能。据报道，S6K1和S6K2在SCLC细胞系中可过度表达，促进肿瘤细胞增殖。由于其各自的活化不依赖PI3K，于是可被特异性mTOR抑制剂阻断^[28]^。mTOR抑制剂依维莫司联合标准EP方案一线治疗转移性SCLC正在研究中。

### 2.2 成纤维细胞生长因子受体-1（fibroblast growth factor receptor-1, FGFR1）扩增

FGFR1由位于染色体8p12上的独立基因编码。其与配体FGFs结合后，主要通过激活下游RAS/RAf/MEK/Erk、PI3K/Akt信号通路，参与调节胚胎发育、组织修复、血管生成等重要过程。FGR-FGFR信号转导通路异常在多种肿瘤的生长、移行和血管生成过程中起核心作用。

据报道^[29]^，大约20%肺鳞癌中存在*FGFR1*基因扩增，以FGFR1为靶点的分子药物治疗已进入临床试验阶段，并在肺鳞癌患者中取得一定疗效。Schultheis等^[30]^应用荧光原位杂交（fluorescence *in situ* hybridization, FISH）法检测251份SCLC肿瘤组织，证实约5.6%的SCLC也存在高水平FGFR1扩增，但与肺鳞癌扩增形式有显著不同，主要为同源性扩增，且均匀分布于肿瘤细胞，无肺鳞癌中的“分布热点”现象。Ruotsalainen等^[31]^在刚确诊SCLC患者的血清中发现存在高水平FGF-2，既往研究证明FGF-2与肿瘤血管生长丰富及预后不良密切相关。Pardo等^[32]^研究发现FGF-2可作为SCLC细胞的促分离原，并诱导凋亡蛋白抑制因子Bcl-XL、XIAP表达，逃避依托泊苷诱导的细胞凋亡。FGF/FGFRs抑制剂在SCLC中的有效性值得进一步研究。

### 2.3 *SOX2*扩增

SOX转录因子家族主要参与调节胚胎干细胞及神经干细胞分化，也是肺发育过程中的一类重要功能调节因子。*SOX2*突变与*FGFR1*突变一样，在肺鳞癌多见。*SOX*扩增在SCLC中占27%，Titulaer等^[33]^应用shRNA沉默SOX2表达后存在*SOX2*扩增的SCLC细胞系明显生长受抑。

### 2.4 *MYC*扩增

原癌基因*MYC*转录产物具有刺激细胞增殖和促进细胞凋亡双重作用，与恶性肿瘤形成、发展有密切关系。*L-MYC*、*N-MYC*、*C-MYC*异常扩增在肺癌中较明确，其中*L-MYC*、*N-MYC*在SCLC中更多见。有研究^[34]^显示SCLC中*MYC*扩增一般伴有抑癌基因*MAX*（MYC-associated factor X gene）、*BRG1*基因的失活，而并未在NSCLC中观察到此现象。研究显示*MYC*突变可能发生在癌变过程的启动阶段。

### 2.5 EGFR

EGFR属于Ⅰ酪氨酸激酶型受体，激活后触发肿瘤细胞增殖、分化、迁移、粘附、血管形成等一系列下游事件。除已知肺鳞癌和肺腺癌中存在EGFR过度表达，也有证据表明在SCLC中存在低水平的*EGFR*突变，且存在*EGFR*突变的SCLC细胞系表现出更强的侵袭性。*EGFR*突变阳性率水平与其对酪氨酸激酶抑制剂（tyrosine kinase inhibitor, TKI）类药物的敏感性并不是完全平行的。体外试验中SCLC细胞系对吉非替尼表现出一定敏感性。但之后的Ⅱ期临床试验并未观察到有效反应。有个例报道TKI类药物在广泛期SCLC有一定临床效果。

### 2.6 c-Kit

人类*c-Kit*原癌基因位于人染色体4qll-12位点上，其mRNA长约5, 084 bp，其中2, 928 bp编码蛋白产物，即为c-Kit受体（CD117）。该受体为Ⅲ型酪氨酸激酶受体，与配体干细胞因子（stem cell factor, SCF）结合，激活下游信号转导通路，包括P13K通路、JAK -STAT通路、MAPK通路等，促进各类细胞的增殖、迁移和分化^[35]^。

L’opez-Martin等^[36]^利用免疫组化方法分析204例SCLC标本，结果显示73%（*n*=149）有c-Kit表达。在离体实验中，c-Kit阳性SCLC细胞系接触到SCF后瘤细胞可出现迅速增殖及转移，抑制c-Kit受体后SCLC肿瘤细胞的生长则明显受限^[37, 38]^。但在进一步的临床试验中，c-Kit抑制剂如伊马替尼却未能在SCLC患者中显示抗肿瘤活性。

### 2.7 c-Met

c-Met属于Ⅱ型酪氨酸激酶受体，配体为肝细胞生长因子（human hepatocyte growth factor, HGF）。c-Met/HGF信号转导途径活化后可以促进肿瘤细胞增殖、运动、分化及血管形成，对肿瘤的侵袭和转移有重要促进作用。c-Met高表达在多种恶性肿瘤中已有报道，在各型肺癌中以SCLC多见。Wang等^[39]^运用siRNA下调c-Met表达后发现SCLC细胞增殖性以及侵袭性均明显受抑制，表明c-Met/HGF信号转导途径异常在SCLC的形成及进展中起重要作用。多种c-MET抑制剂如AMG102联合标准EP方案（依托泊苷+顺铂）治疗转移性SCLC的临床研究正在进行中。

SCLC中已知许多致癌基因突变及分子改变，早期进行的针对其特异性靶点分子靶向治疗药物，如VEGFR抑制剂、bcl-2反义寡核苷酸、IGF-IR抑制剂等，在进行的多项临床试验中并没有明显改变本病的进程。最新研究表明EZH2、PARP-1在SCLC中存在明显高表达现象，特异性优于其他分子治疗靶点，有望由此在SCLC靶向治疗方面获得新的突破。但是，目前以EZH2、PARP-1为靶点的分子靶向治疗在SCLC中开展的研究性试验还十分有限，EZH2、PARP-1具体的致癌分子作用机制仍需进一步研究，相应药物的研发及其在SCLC治疗中的敏感性及安全性问题更需要未来很长一段时间逐步逐层论证落实。此外，在一些SCLC发生发展中可能存在数个驱动基因或信号通路同时异常活化，各自通过不同机制促进肿瘤形成，也许是单一分子靶向治疗效果不佳原因之一。总而言之，目前仍然需要对SCLC驱动基因及具体分子作用机制不断深入研究，以指导临床优化治疗策略。

## References

[1] Pietanza MC, Ladanyi M (2012). Bringing the genomic landscape of small-cell lung cancer into focus. Nat Genet.

[2] Peifer M, Fernández-Cuesta L, Sos ML (2012). Integrative genome analyses identify key somatic driver mutations of small-cell lung cancer. Nat Genet.

[3] Meuwissen R, Linn SC, Linnoila RI (2003). Induction of small cell lung cancer by somatic inactivation of both Trp53 and Rb1 in a conditional mouse model. Cancer Cell.

[4] Rudin CM, Durinck S, Stawiski EW (2012). Comprehensive genomic analysis identifies SOX2 as a frequently amplified gene in small-cell lung cancer. Nat Genet.

[5] Iwakawa R, Takenaka M, Kohno T (2013). Genome-wide identification of genes with amplification and/or fusion in small cell lung cancer. Genes Chromosomes Cancer.

[6] Byers LA, Wang J, Nilsson MB (2012). Proteomic profiling identifies dysregulated pathways in small cell lung cancer and novel therapeutic targets including PARP1. Cancer Discov.

[7] Wistuba Ⅱ, Gazdar AF, Minna JD (2001). Molecular genetics of small cell lung carcinoma. Semin Oncol.

[8] Coe BP, Thu KL, Aviel-Ronen S (2013). Genomic deregulation of the E2F/Rb pathway leads to activation of the oncogene EZH2 in small cell lung cancer. PLoS One.

[9] Zacharatos P, Kotsinas A, Evangelou K (2004). Distinct expression patterns of the transcription factor E2F-1 in relation to tumour growth parameters in common human carcinomas. J Pathol.

[10] Vissers JH, van Lohuizen M, Citterio E (2012). The emerging role of Polycomb repressors in the response to DNA damage. J Cell Sci.

[11] Tiffen J1, Gallagher SJ, Hersey P (2015). EZH2: an emerging role in melanoma biology and strategies for targeted therapy. Pigment Cell Melanoma Res.

[12] Simon JA, Lange CA (2008). Roles of the EZH2 histone methyltransferase in cancer epigenetics. Mutat Res.

[13] Hubaux R, Thu KL, Coe BP (2013). EZH2 promotes E2F-driven SCLC tumorigenesis through modulation of apoptosis and cell-cycle regulation. J Thorac Oncol.

[14] Bryant RJ, Cross NA, Eaton CL (2007). EZH2 promotes proliferation and invasiveness of prostate cancer cells. Prostate.

[15] Kleer CG, Cao Q, Varambally S (2003). EZH2 is a marker of aggressive breast cancer and promotes neoplastic transformation of breast epithelial cells. Proc Natl Acad Sci USA.

[16] Arisan S, Buyuktuncer ED, Palavan-Unsal N (2005). Increased expression of EZH2, a polycomb group protein, in bladder carcinoma. Urol Int.

[17] Mathews LA, Crea F, Farrar WL (2009). Epigenetic gene regulation in stem cells and correlation to cancer. Differentiation.

[18] Hu S, Yu L, Li Z (2010). Overexpression of EZH2 contributes to acquired cisplatin resistance in ovarian cancer cells *in vitro* and *in vivo*. Cancer Biol Ther.

[19] Vyas S, Chang P (2014). New PARP targets for cancer therapy. Nat Rev Cancer.

[20] Woodhouse BC, Dianov GL (2008). Poly ADP-ribose polymerase-1: An international molecule of mystery. DNA Repair.

[21] Tallis M1, Morra R, Barkauskaite E (2014). Poly(ADP-ribosyl)ation in regulation of chromatin structure and the DNA damage response. Chromosoma.

[22] Hegan DC, Lu Y, Stachelek GC (2010). Inhibition of poly (ADP-ribose) polymerase down-regulates BRCA1 and RAD51 in a pathway mediated by E2F4 and p130. Proc Natl Acad Sci U S A.

[23] Cardnell RJ, Feng Y, Diao L (2013). Proteomic markers of DNA repair and PI3K pathway activation predict response to the PARP inhibitor BMN 673 in small cell lung cancer. Clin Cancer Res.

[24] Juvekar A, Burga LN, Hu H (2012). Combining a PI3K inhibitor with a PARP inhibitor provides an effective therapy for BRCA1-related breast cancer. Cancer Discov.

[25] Yokomizo A, Tindall DJ, Drabkin H (1998). *PTEN*/*MMAC1* mutations identified in small cell, but not in non-small cell lung cancers. Oncogene.

[26] Shibata T, Kokubu A, Tsuta K (2009). Oncogenic mutation of PIK3CA in small cell lung carcinoma: a potential therapeutic target pathway for chemotherapy-resistant lung cancer. Cancer Lett.

[27] Moore SM, Rintoul RC, Walker TR (1998). The presence of a constitutively active phosphoinositide 3-kinase in small cell lung cancer cells mediates anchorage-independent proliferation via a protein kinase B and p70s6k-dependent pathway. Cancer Res.

[28] Pardo OE, Arcaro A, Salerno G (2001). Novel cross talk between MEK and S6K2 in FGF-2 induced proliferation of SCLC cells. Oncogene.

[29] Weiss J, Sos ML (2010). Frequent and focal FGFR1 amplification associates with therapeutically tractable FGFR1 dependency in squamous cell lung cancer. Sci Transl Med.

[30] Schultheis AM, Bos M, Schmitz K (2014). Fibroblast growth factor receptor 1 (FGFR1) amplification is a potential therapeutic target in small-cell lung cancer. Mod Pathol.

[31] Ruotsalainen T, Joensuu H, Mattson K (2002). High pretreatment serum concentration of basic fibroblast growth factor is a predictor of poor prognosis in small cell lung cancer. Cancer Epidemiol Biomarkers Prev.

[32] Pardo OE, Arcaro A, Salerno G (2002). Fibroblast growth factor-2 induces translational regulation of Bcl-XL and Bcl-2 via a MEK-dependent pathway: correlation with resistance to etoposide-induced apoptosis. J Biol Chem.

[33] Titulaer MJ, Klooster R, Potman M (2009). SOX antibodies in small-cell lung cancer and Lambert-Eaton myasthenic syndrome: frequency and relation with survival. J Clin Oncol.

[34] Romero OA, Torres-Diz M, Pros E (2014). MAX inactivation in small cell lung cancer disrupts MYC-SWI/SNF programs and is synthetic lethal with BRG1. Cancer Discov.

[35] Heinrich MC, Blanke CD, Druker BJ (2002). Inhibition of KIT tyrosine kinase activity: a novel molecular approach to the treatment of KIT-positive malignancies. J Clin Oncol.

[36] López-Martin A, Ballestín C, Garcia-Carbonero R (2007). Prognostic value of KIT expression in small cell lung cancer. Lung Cancer.

[37] Krystal GW, Hines SJ, Organ CP (1996). Autocrine growth of small cell lung cancer mediated by coexpression of c-kit and stem cell factor. Cancer Res.

[38] Yamanishi Y, Maeda H, Hiyama K (1996). Specific growth inhibition of small-cell lung cancer cells by adenovirus vector expressing antisense c-kit transcripts. Jpn J Cancer Res.

[39] Wang ZX, Lu BB, Yang JS (2011). Adenovirus-mediated siRNA targeting c-Met inhibits proliferation and invasion of small-cell lung cancer (SCLC) cells. J Surg Res.

